# Verbal and Nonverbal Memory in Primary Progressive Aphasia: The Three Words-Three Shapes Test

**DOI:** 10.3233/BEN-2012-110239

**Published:** 2013

**Authors:** Sandra Weintraub, Emily Rogalski, Emily Shaw, Sabrina Sawlani, Alfred Rademaker, Christina Wieneke, M.-Marsel Mesulam

**Affiliations:** ^1^Cognitive Neurology and Alzheimer's Disease CenterNorthwestern University Feinberg School of MedicineChicagoILUSA; ^2^Department of Psychiatry and Behavioral SciencesNorthwestern University Feinberg School of MedicineChicagoILUSA; ^3^Department of Preventive MedicineNorthwestern University Feinberg School of MedicineChicagoILUSA; ^4^Ken and Ruth Davee Department of NeurologyNorthwestern University Feinberg School of MedicineChicagoILUSA

## Abstract

*Objectives:* To investigate cognitive components and mechanisms of learning and memory in primary progressive aphasia (PPA) using a simple clinical measure, the Three Words Three Shapes Test (3W3S).

*Background:* PPA patients can complain of memory loss and may perform poorly in standard tests of memory. The extent to which these signs and symptoms reflect dysfunction of the left hemisphere language versus limbic memory network remains unknown.

*Methods:* 3W3S data from 26 patients with a clinical diagnosis of PPA were compared with previously published data from patients with typical dementia of the Alzheimer type (DAT) and cognitively healthy elders.

*Results:* PPA patients showed two bottlenecks in new learning. First, they were impaired in the effortless (but not effortful) on-line encoding of verbal (but not non-verbal) items. Second, they were impaired in the retrieval (but not retention) of verbal (but not non-verbal) items. In contrast, DAT patients had impairments also in effortful on-line encoding and retention of verbal and nonverbal items.

*Conclusions:* PPA selectively interferes with spontaneous on-line encoding and subsequent retrieval of verbal information. This combination may underlie poor memory test performance and is likely to reflect the dysfunction of the left hemisphere language rather than medial temporal memory network.

## 1. Introduction

The diagnosis of PPA is based on the *presence* of aphasia in the earliest stages of illness but also on the *absence* of impairment in other cognitive domains, including episodic memory, reasoning, and visuospatial functions [1–5]. However, the clinician is frequently faced with patients and families who report "memory loss" even though there is usually little indication that daily living activities are being undermined by impaired episodic memory. Is the reported 'memory' impairment a secondary manifestation of the aphasia or is there an amnestic component in PPA? This is a challenging question to address through neuropsychological assessment because many of the relevant tasks require verbal instructions, contain verbal stimuli and/or require a verbal response. For example, one of the

ISSN 0953-4180/13/$27.50 © 2013 – IOS Press and the authors. All rights reserved most common tests of dementia severity, the Mini Mental State Examination (MMSE) [6] relies heavily on language processing. Thus, although the MMSE has served to characterize severity in a variety of clinical dementia syndromes where language is not the primary deficit, it may overestimate the extent of functional impairment in PPA. This dissociation between test scores and daily function was demonstrated in a study comparing patients with PPA, patients with dementia of the Alzheimer type (DAT) and patients with behavioral variant frontotemporal dementia on the Activities of Daily Living Questionnaire (ADLQ) [7], an informant completed assessment of activities of daily living. Despite similar MMSE scores in the three groups, the PPA group scored consistently better on subscales of the ADLQ than the other two groups. The relative preservation of episodic memory and executive function in the PPA patients likely accounted for their ability to function at a higher level, despite the language impairment [8]. Thus, the principled assessment of memory and learning in PPA has important implications for the rigorous confirmation of the diagnosis and also for the comprehensive assessment of functional capacity.

The ability to recall an experience requires the perceptual encoding and on-line holding of the constituent information, its subsequent transformation into distributed off-line neural representations that sustain long-term retention, and the ability for the binding and coherent reactivation of these representations during voluntary recall. The mediotemporal limbic system plays its pivotal role in episodic memory by binding and then coherently activating distributed information pertaining to recent multimodal events [9]. This system is not a primary target of pathology in PPA. However, some of the distributed information belonging to recent experience, such as modality-specific word-forms and object representations, are encoded in perisylvian and inferotemporal areas that do become the targets of initial atrophy in PPA. It is therefore reasonable to assume that PPA may be associated with material- and modality-specific impairments of new learning despite the overall preservation of episodic memory for recent experiences. A selective loss of verbal memory is frequently mentioned in the literature on PPA but is rarely documented with parallel verbal and non-verbal tasks. The current study was undertaken to investigate if memory and learning for visually presented words and shapes are affected differently in PPA and, if so, whether this dissociation encompasses all or only some of the component processing stages.

We had designed a six-item memory test, the Three Words Three Shapes Test (3W3S), to specifically assess verbal and non-verbal memory as well as the constituent stages of perceptuomotor encoding, on-line holding, retention, recall, retrieval and recognition [10]. A systematic scoring method was subsequently devised so that the 3W3S test could be used to discriminate between age-related memory loss and amnesia associated with DAT [11]. The 3W3S Test was designed to fulfill the need for a simple test that could be used in moderate stages of illness so that patients would not show the floor effects that are common when using most standard memory measures [12]. In addition, there were several unique attributes built into the test. First, most memory tests confound material (verbal vs nonverbal) with modality (auditory vs visual). For example, verbal memory is most often tested in the auditory modality with oral word lists or stories, while non verbal memory is tested in the visual modality by reproduction of geometric designs or picture recognition. In order to keep the modality consistent, words and shapes were both presented as visual stimuli in the 3W3S Test. Secondly, we required that all stimuli be copied prior to assessing memory to ensure that there were no primary writing or drawing deficits that could affect subsequent recall performance. A third innovation was to have an incidental encoding condition. Thus, participants were not forewarned to remember the stimuli as they copied them and recall was tested immediately after copying. This condition assesses the amount of information that can be encoded without effort, a type of memory that has greater ecological validity than memory based on rote learning. Following incidental recall of the six items, a number of effortful encoding (i.e., rote learning) trials were presented to bring the participant to a criterion level before testing delayed recall. Delayed recall was then tested and compared to the recognition of the items in order to assess the specific contribution of retrieval versus retention failure to overall explicit memory.

## 2. Methods

### 2.1. Study design

The study design was a retrospective analysis of test performance on the Three Words Three Shapes test in the context of a clinical evaluation for PPA. Data from the PPA participants were compared with previously published data from individuals with DAT and cognitively healthy elders.

Table 1
Cognitive operations indexed by 3W3S scores

| | |
|---|---|
| Copy | Perceptuomotor aspects of the task |
| Incidental encoding | Capacity for effortless, spontaneous encoding of information and its on-line holding for immediate reproduction |
| Effortful encoding | Capacity for effortful, deliberate encoding of information and its on-line holding for immediate reproduction |
| Delayed recall | Amount of information that has been transferred to and retrieved from off-line storage |
| Recognition | Amount of information that has been transferred to off-line storage and retained in a form that can sustain recognition |
| Difference between effortful encoding and delayed recall | Represents the amount of information that fails to be transferred from on-line to off-line storage in a form that can be retained and reproduced |
| Difference between recognition and delayed recall | Represents the memory impairment that can be attributed to a deficit of retrieval rather than retention |

### 2.2. Subjects

The sample consisted of 26 patients participating in a research project on PPA at the Northwestern University Cognitive Neurology and Alzheimer's Disease Center (DNADC). Informed consent was obtained on a research protocol approved by the Institutional Review Board at Northwestern University to participate in a three-day study, which included neuropsychological testing, experimental tests of language processing and structural neuroimaging. They had agreed to review of their clinical records with HIPAA assurances for information pertinent to their condition and participation in research. Comparison data from the previously published study had been obtained on 21 patients with a diagnosis of DAT and 14 cognitively normal controls.

All PPA patients had been given a root diagnosis of PPA [1,3,4] based on clinical evaluation in a memory disorders clinic. In addition, all patients had been subtyped on the basis of clinical impression on examination. Some had also been subtyped on the basis of an algorithm developed for this purpose based on measures of fluency, single word comprehension, naming and grammatical competence [13] and in all cases this subtyping was in agreement with the clinical assessment. One group was designated as agrammatic (PPA-G, N = 10) based on prominent deficits in grammatical processing and preserved single word comprehension and naming; a second group was designated as logopenic (PPA-L, N = 7) based on preserved grammatical processing and single word comprehension and reduced speech fluency and naming; a third group was labeled semantic (PPA-S, N=7) on the basis of impaired single word comprehension and naming and preserved grammar. Two patients could not be classified because the severity of their symptoms resulted in deficits in multiple components of language processing.

### 2.3. Materials and procedures

3W3S had been administered to participants as part of their evaluation in a behavioral neurology clinic. As previously described [11], the stimuli consist of three simple geometric designs and three words, one under each of the designs, horizontally distributed on a letter-sized page, in portrait orientation. (Test and scoring forms can be downloaded from our website www.brain.northwestern.edu).

Table 1 lists the conditions of the 3W3S test and the corresponding operations each measures. Patients are first asked to copy the three designs and the words directly beneath the stimuli. Participants are not forewarned to remember the stimuli but immediately after copying them, the page is removed and they are given a blank sheet and asked to reproduce what they just copied (Incidental Encoding trial). If the patient remembers at least five of the six stimuli, Delayed Recall is tested as described below. If on the Incidental Encoding trial the subject recalls fewer than five stimuli, the original stimulus sheet is presented for a 30-second study with the explicit instruction to try to remember the six stimuli in order to reproduce them from memory (Effortful Encoding trial). If after the first such trial the subject recalls five of the six stimuli, Delayed Recall is tested (below). If fewer than five stimuli are reproduced correctly, these trials are repeated up to a total of five or until five of the six stimulus items are reproduced correctly. Figure 1 shows a sample from a patient (number 17, Table 2).

Delayed recall was tested once, following either Incidental Encoding if criterion had been reached at that point, or following the last Effortful Encoding trial. Because of the constraints of the clinical examination, delay intervals ranged from 10 to 40 minutes, with a mean of 19.92 $\pm$ 5.91 minutes (median and mode = 20). Multiple choice recognition was tested following

Table 2
Demographics and clinical test scores of PPA sample

| Subject ID | Gender | Subtype | Education years | Age at visit | Symptom duration (Years) | WAB AQ 100 | WAB Rep 100 | BNT score 60 | PPVT 36 | NAT 10 | CDR sum boxes | Global CDR score |
|---|---|---|---|---|---|---|---|---|---|---|---|---|
| 1 | Male | NS/L | 15 | 70 | 2 | 92 | 90 | 59 | 35 | 9 | 0 | 0 |
| 2 | Male | NS/G | 15 | 64 | 8.5 | – | – | – | – | – | – | – |
| 3 | Male | NS/L | 18 | 63 | 2 | 97.1 | 89 | 58 | 36 | 10 | 0.5 | 0 |
| 4 | Female | Severe | 12 | 63 | 4 | 41.2 | 32 | 9 | NA | | 2.5 | 0.5 |
| 5 | Female | NS/L | 18 | 62 | 3 | 88.7 | 95 | 22 | ND | ND | 1.5 | 0.5 |
| 6 | Male | NS/G | 12 | 58 | 2 | – | – | – | – | – | – | – |
| 7 | Female | NS/L | 16 | 74 | 2 | – | – | 49 | – | – | – | – |
| 8 | Female | Semantic | 12 | 54 | 3.5 | 82.4 | 97 | 8 | – | – | 1 | 0.5 |
| 9 | Male | Semantic | 20 | 64 | 5 | 88.2 | 92 | 14 | 31 | 9 | 1 | 0.5 |
| 10 | Female | NS/L | 16 | 78 | 4.5 | 69.5 | 54 | 10 | 32 | 9 | 0 | 0 |
| 11 | Male | NS/G | 17 | 54 | 4 | 50 | 72 | 4 | 32 | 1 | 2.5 | 0.5 |
| 12 | Female | NS/G | 16 | 64 | 5 | 38.6 | 19 | 3 | 25 | – | – | – |
| 13 | Female | NS/L | 14 | 78 | 3.5 | 62.6 | 50 | 39 | 29 | – | 1.5 | 0.5 |
| 14 | Male | NS/G | 16 | 70 | 3.5 | 78.1 | 74 | 43 | 33 | 4 | 1 | 0.5 |
| 15 | Female | NS/G | 14 | 81 | 2 | 93.7 | 90 | 56 | – | – | 1.5 | 0.5 |
| 16 | Male | NS/G | 13 | 76 | 4 | 71.6 | 78 | 50 | ND | ND | 1.5 | 0.5 |
| 17 | Female | Semantic | 16 | 52 | 2.5 | – | – | – | – | – | – | – |
| 18 | Male | Semantic | 20 | 64 | 2 | 87.4 | 97 | 6 | 31 | 10 | 1 | 0.5 |
| 19 | Female | NS/G | 19 | 62 | 3 | 76.2 | 60 | 24 | 35 | 6 | 1 | 0 |
| 20 | Male | NS/G | 14 | 57 | 4 | 77.7 | 92 | 44 | 28 | 4 | 1.5 | 0.5 |
| 21 | Female | NS/L | 18 | 64 | 1.5 | 83.2 | 95 | 59 | 33 | 8 | 0 | 0 |
| 22 | Male | Semantic | 15 | 60 | 4 | – | – | – | – | – | – | – |
| 23 | Female | Semantic | 18 | 56 | 2 | 75.5 | 84 | 3 | 14 | 10 | 3 | 0.5 |
| 24 | Female | Semantic | 16 | 56 | 4 | – | – | 16 | – | – | 4 | 1 |
| 25 | Female | NS/G | 18 | 57 | 4 | 76.7 | 70 | 41 | 34 | 5 | 3.5 | 0.5 |
| 26 | Male | Severe | 14 | 58 | 4 | 60.3 | 45 | 20 | – | – | 2 | 0.5 |
| | | **MEAN** | **15.84** | **63.81** | **3.44** | **74.54** | **73.75** | **28.95** | **30.57** | **7.08** | **1.53** | **0.40** |
| | | *St Dev* | *2.34* | *8.16* | *1.47* | *16.68* | *23.24* | *20.85* | *5.63* | *3.00* | *1.12* | *0.26* |
| | | *N* | | | | *20.00* | *20.00* | *22.00* | *14.00* | *12.00* | *20.00* | *20.00* |

WAB = Western Aphasia Battery-Revised; AQ = Aphasia Quotient; Rep = Repetition; BNT = Boston Naming Test; PPVT-Peabody Picture Vocabulary Test; NAT = Northwestern Anagram Test; CDR = Clinical Dementia Rating; NS = Non semantic PPA subtype; G = agrammatic subtype; L = logopenic subtype; St Dev: standard deviation; N = number of observations on which means and standard deviations are based for each clinical measure.

the delay trial, unless the patient spontaneously and accurately recalled the stimuli. Nineteen of the patients were administered the Recognition condition; the rest had reproduced that stimuli correctly on delayed recall. They were asked to select the three words and three designs that they recalled from the test from among a set of 10 words and 10 designs.

Each condition was scored using previously developed criteria [11]. Each word had a total possible score of 5, 15 points for all the words; each design had a total score of 5, 15 for all the designs. Inter-rater reliability was established for the scoring criteria. Two raters were trained to use the scoring criteria. Each coded all 26 test protocols. A comparison of all scoring points for each condition indicated 100% agreement for the words. For the shapes the Kappa coefficient was 0.84, considered a level of almost perfect agreement. In instances where there was disagreement, however, the scores were discussed and consensus achieved.

### 2.4. Analyses

Analyses were designed to compare the PPA, DAT and control groups with respect to the components of verbal and nonverbal episodic memory assessed with 3W3S. Of particular interest was the comparison between effortful encoding and delayed recall, which would reflect the extent to which retrieval was affected, and the comparison between delayed recall and recognition which would provide information about the retention of information over time (Table 1). These distinctions were tested with ANOVA comparing performance of the PPA group with prior data derived from patients with DAT and cognitively healthy controls previously reported [11]. In the original 3W3S study, participants were tested at three delay intervals, five-, 15-, and 30-minutes following either the incidental encoding or the last Effortful Encoding trial. Results had shown that there was a significant reduction in the

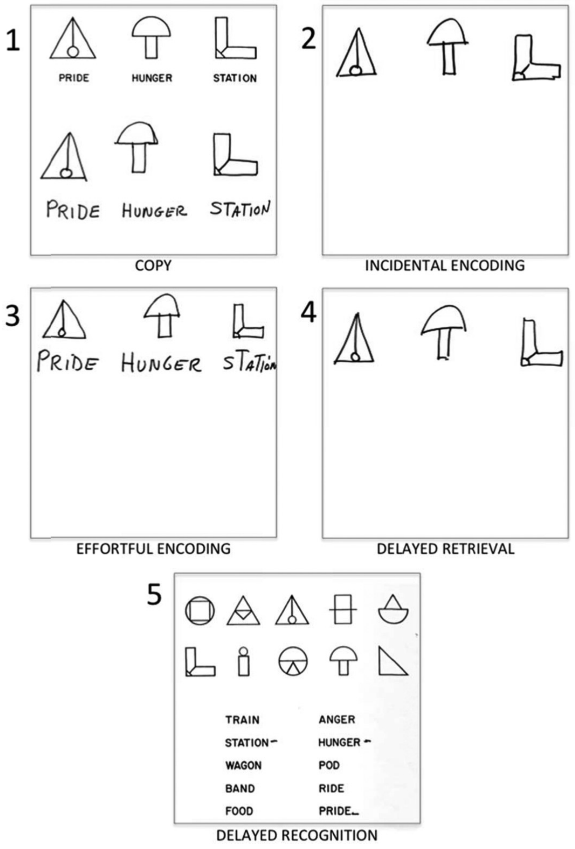

Fig. 1. Sample of performance from a patient with primary progressive aphasia (number 17, Table 2). 1) Copy. The patient was able to accurately copy all of the six stimuli. 2) Incidental encoding trial. The patient correctly reproduced all the shapes but none of the words. 3) Effortful encoding. With only one additional exposure and the instruction to remember all the stimuli, the patient could reproduce all the shapes and the words. 4) Delayed retrieval. After 10 minutes, the patient recalled all the shapes and none of the words. 5) Delayed recognition. The recognition form contains 10 shapes and 10 words, including the original 6 stimuli. Immediately after the delayed recall condition, in which she obtained a perfect score for shapes but could not recall any of the words, she recognized the three words.

amount of information retrieved after the initial delay of five minutes with no further loss over remaining delay intervals [11]. For purposes of comparison with data from the present study, the 15-minute delayed recall condition was used from the original cohort since it was closest in duration to our 20-minute average for the PPA participants.

Two additional ANOVA's were conducted within the PPA group to compare verbal and nonverbal memory: one on the entire group of PPA patients and a second comparing the three PPA subtypes. Two patients were too impaired to be subtyped (see below) and were excluded from this analysis.

## 3. Results

### 3.1. Demographics and clinical tests

Table 2 shows individual PPA participants' demographics and clinical test scores. Because of the retrospective nature of the study, not all individuals in the PPA group were administered the same battery of clinical tests at the same time as they were tested with 3W3S. Table 2 indicates the numbers of subjects on which each mean score is based.

Of the twenty-six patients included in this study 12 were male and 14 female. The average age was 63.81 ± 8.16 years and average education level was 15.85 ± 2.34 years. There was a large age range from 52 to 81 years. In the PPA group, symptom duration varied from 1.5 to 8.5 years with a mean of 3.44 ± 1.47 years. Seven of the PPA participants were subtyped as semantic, 17 as non semantic (10 agrammatic and 7 logopenic) and two could not be subtyped (Table 2, subjects 4 and 26) due to the severe nature of their condition resulting in more generalized language deficits. The previously published DAT and control samples were somewhat older (DAT mean = 66.38 ± 3.10 years; control mean = 70.86 ± 2.20 years) and less well educated (DAT mean education = 12.90 ± 0.51 years; control mean education = 13.79 ± 0.68 years). Dementia severity, as measured by the Blessed Dementia Scale [14] was within the mild-moderate range (mean = 10.03 ± 1.07, of a possible 37; higher score = greater impairment) similar to the aphasia severity range for the PPA sample.

The average WAB AQ, a measure of aphasia severity was 74.54 ± 16.68, indicating a moderate level overall, but considerable variability. Scores ranged from 41.2 (severely impaired) to 97.2 (very mildly impaired). Although the average score on the Boston Naming Test was very low (28.95 ± 20.85) the range was from 3 to 59. Scores on the Peabody Picture Vocabulary Test (36 items) ranged from 14 to 36, mean = 30.57 ± 5.63. Only 14 participants had been administered the 10-item form of the Northwestern Anagram Test (NAT-10) a measure of grammatical processing and the average score was 7.08 ± 3.00.

Dementia severity was measured by the mean Global Score on the Clinical Dementia Rating (CDR) scale [15] and was 0.40 ± 0.26 (very mildly impaired). The CDR Sum of Boxes score [16], which provides an expanded scoring range for the CDR, also indicated a very minimal level of dementia severity (mean = 1.53 ± 1.12). In early stages, patients with PPA typically do not have the memory and orientation deficits that figure prominently in dementia of the Alzheimer type and that are the focus of the CDR. The CDR is also based on the level of impairment of judgment and problem solving, functioning in home and hobbies, and ability in community affairs. Patients with PPA, aside from their aphasia, do not have early impairments in these areas. Thus, despite the range of aphasia severity, all the patients in the sample were able to perform the 3W3S.

### 3.2. *Three words three shapes performance*

#### 3.2.1. *Comparison among study groups*

Table 3 shows mean scores and standard deviations of 3W3S performance by condition for the PPA, DAT and healthy control groups. As previously reported, elderly controls perform near ceiling on all components of this test, explained by our effort to design an instrument with a reduced level of difficulty for patients with dementia. The results are presented as they address each of the episodic memory components measured. Significance levels were interpreted with Bonferroni corrections for multiple pairwise comparisons ($p = 0.016$).

#### 3.2.2. *Perceptuomotor components*

There were no significant differences between PPA, DAT and healthy controls on the Copy condition, indicating that perceptuomotor components of the task were sufficient to direct attention to and support adequate rendering of the stimuli. Thus, low scores in subsequent conditions could not be explained by more primary perceptuomotor dysfunction.

#### 3.2.3. *Incidental encoding*

This condition represents the capacity for online spontaneous and effortless encoding for immediate recall. Under this condition, the PPA group's performance was worse than controls for words ($p < 0.001$) but similar to DAT patients ($p = 0.37$) (mean PPA = $4.63 \pm 4.78$; mean NC = $12.14 \pm 3.23$; mean DAT = $5.95 \pm 6.59$). In contrast, their Incidental Encoding score for shapes was not significantly different from that of controls ($p = 0.062$) and superior to that of DAT patients ($p < 0.0001$) (mean PPA = $8.88 \pm 4.77$; mean NC = $11.79 \pm 3.56$; mean DAT = $4.33 \pm 2.37$.

#### 3.2.4. *Effortful encoding*

With the explicit instruction to commit the stimuli to memory, that is to engage in effortful encoding, PPA patients required more trials than NC (mean = $1.96 \pm 1.00$ vs $0.43 \pm 0.51$; $p = 0.0002$) but fewer trials than DAT patients (mean = $3.10 \pm 1.58$, $p = 0.0015$) to reach criterion. At the last acquisition trial the three groups did not differ in their scores for words. However effortful encoding of shapes was similar in PPA and control groups and both groups had significantly higher scores for shapes than the DAT group (both $p < 0.0001$). These results indicate that after a few additional trials, PPA patients are able to immediately retrieve words and shapes at a level similar to controls while patients with DAT have more difficulty with immediate retrieval of shapes than words.

#### 3.2.5. *Delayed retrieval*

Following the delay interval, PPA patients had lower scores than controls for word recall (mean PPA = $7.79 \pm 5.84$ vs mean controls = $13.43 \pm 2.62$, $p = 0.0004$) but similar scores to controls for shape recall (mean PPA = $12.29 \pm 3.34$; mean controls = $14.29 \pm 1.38$, $p = 0.18$). However, PPA patients had higher scores for both words and shapes than DAT patients (mean DAT Words = $2.05 \pm 3.43$; Shapes = $5.05 \pm 6.16$; both $p < 0.0001$). These results imply that there is a material specific effect for word retrieval after a delay in PPA when compared with cognitively healthy controls but superior performance to patients with amnestic DAT regardless of material.

#### 3.2.6. *Delayed recognition*

On multiple choice recognition, a measure of retentive memory, PPA patients obtained near ceiling scores for both words and shapes, better than DAT patients ($p < 0.0001$, 0.0014, respectively), and similar to controls ($p = 0.65$, 0.96, respectively).

#### 3.2.7. *Within-group analysis of all PPA patients*

ANOVA was conducted to identify material specific (words and shapes) and task components (i.e., copy, recall) deficits within the PPA group. PPA patients' scores for shapes was higher than for words on the incidental and delayed recall conditions ($p = 0.0004$, 0.0015, respectively, Table 3). Words and shapes scores were similar on all the other conditions, namely, copy, effortful encoding, and delayed recognition. These results indicate that when retrieval is aided by effortful encoding or by the absence of a delay, retentive memory is as good for words as it is for shapes.

Three within group comparisons were of particular interest for identifying the bottleneck for performance in the PPA patients. First, comparing the last effort-

Table 3
3W3S scores for PPA, DAT, NC

| Subject group | Copy W | Copy S | Incidental recall W | Incidental recall S | Last acquisition W | Last acquisition S | Delayed recall** W | Delayed recall** S | Recognition W | Recognition S | Effortful encoding trials (#) |
|---|---|---|---|---|---|---|---|---|---|---|---|
| PPA (N = 26) | | | | | | | | | | | |
| Mean | 14.96 | 14.83 | 4.63 | 8.88 | 12.57 | 13.24 | 7.79 | 12.29 | 14.25 | 14.93 | 1.96 |
| *SD* | *0.20* | *0.48* | *4.78* | *4.77* | *4.08* | *2.45* | *5.84* | *3.34* | *1.29* | *0.32* | *1.00* |
| DAT (N = 21) | | | | | | | | | | | |
| Mean | 15.00 | 14.81 | 5.95 | 4.33 | 13.00 | 7.95 | 2.05 | 5.05 | 5.28 | 11.11 | 3.10 |
| *SD* | *0.00* | *0.33* | *6.59* | *2.37* | *3.95* | *4.60* | *3.43* | *6.16* | *6.30* | *6.08* | *1.58* |
| NC (N = 14) | | | | | | | | | | | |
| Mean | 15.00 | 14.93 | 12.14 | 11.79 | 14.50 | 14.36 | 13.43 | 14.29 | 13.57 | 15.00 | 0.43 |
| *SD* | *0.00* | *0.27* | *3.23* | *3.56* | *1.40* | *1.39* | *2.62* | *1.38* | *3.63* | *0.00* | *0.51* |

PPA = Primary Progressive Aphasia; DAT = Dementia of the Alzheimer type; NC = Normal Control.
*Data for DAT (aka probable AD) and NC groups taken from Weintraub et al., 2000.
**Delay For PPA = 20.42 minutes (SD = 6.41); Delay for NC and PrAD = 15 minutes.

Table 4
3W3S: Difference scores (shapes minus words) for non semantic and semantic PPA subtypes

| | Non semantic Words mean (sem) | Non semantic Shapes mean | Difference | Semantic Words mean (sem) | Semantic Shapes mean (sem) | Difference | Non semantic vs semantic p-value |
|---|---|---|---|---|---|---|---|
| Copy | 14.94 (0.05) | 14.76 (0.12) | −**0.1765** | 15.00 (0.08) | 15.00 (0.18) | **0.000** | 0.39 |
| Incidental | 6.12 (1.07) | 10.18 (1.14) | **4.0588*** | 1.43 (1.67) | 8.57 (1.78) | **7.1429**** | 0.27 |
| Acquisition | 12.58 (0.71) | 13.29 (0.58) | **0.7059** | 14.86 (1.11) | 13.57 (0.90) | −**1.2857** | 0.31 |
| Delay | 10.71 (1.06) | 12.59 (0.68) | **1.8824** | 2.71 (1.65) | 13.57 (1.06) | **10.8571***** | **0.0011** |
| Recognition | 14.63 (0.30) | 14.91 (0.08) | **0.2816** | 14.57 (0.44) | 15.00 (0.12) | **0.4286** | 0.76 |

Sem = standard error of the mean. $^{*}p = 0.012$; $^{**}p = 0.005$; $^{***}p = 0.0001$.

ful encoding trial (last acquisition) with delayed recall provides information about retrieval failure while a comparison between effortful encoding and recognition provides information about retention failure. There was a statistically significant difference between effortful encoding and delayed recall scores for words ($p = 0.0003$) but not for shapes ($p < 0.038$, Bonferroni correction for multiple pairwise comparisons $= p < 0.005$). There was no significant difference between effortful encoding and delayed recognition scores for words but there was a significant difference for shapes ($p = 0.003$) with recognition superior (Table 3). This implies that retention for words and shapes is preserved despite problems with word retrieval.

The third comparison of interest was between copy and incidental encoding scores to determine what is retained online without conscious effort. In this instance, patients with PPA had lower scores on incidental encoding than on copy for both words and shapes, suggesting that, without the explicit instruction to remember the information, they are not as efficient in retaining both types of material. However, in the analyses reported earlier, comparing PPA and DAT participants, the PPA group outperformed the DAT group for this type of encoding of shapes.

*3.2.8. Comparison of PPA subtypes*

Similar analyses to the one described above for the entire PPA group were conducted separately on the semantic and the non semantic PPA subgroups and both groups were compared. Table 4 shows 3W3S mean scores for both subtypes. Of interest is the fact that when words and shapes were compared across all conditions for the non semantic PPA group, the only comparison that approached significance ($p = 0.012$, Bonferroni correction for multiple comparisons $= p < 0.01$) was a lower score for words than shapes in the incidental encoding condition. For all other test component comparisons, words and shapes scores did not differ significantly. In contrast, comparisons within the semantic group showed significant differences between words and shapes on both incidental encoding and delayed recall conditions ($p = 0.005$, $p = 0.0001$, respectively).

Semantic and non semantic groups were compared in an analysis that contrasted the *difference scores* between words and shapes scores in each condition. Positive numbers reflect worse word than shape performance while negative numbers reflect the reverse and zero implies no difference. In this comparison, the only statistically significant difference was in the delayed recall condition, where the difference between words and shapes was far greater in the semantic than in the non semantic group (mean semantic difference between words and shapes=10.86; mean non semantic difference $= 1.88$, $p = 0.0011$).

## 4. Discussion

The present study investigated episodic memory in patients with primary progressive aphasia (PPA) using the 3W3S Test, a simple test of verbal and non verbal retentive memory that can be used during routine outpatient clinic assessments. Performance was compared to previously published data from patients with amnestic dementia of the Alzheimer type and cognitively healthy controls [11]. The goal of the present study was to determine if explicit memory in PPA patients is influenced by the type of information to be remembered (verbal vs nonverbal) and if there are retrieval and/or retention deficits. To this end, the ability to reproduce words and shapes was tested under conditions of copying, incidental encoding, effortful encoding, delayed recall and recognition, with all stimuli in the visual modality.

The PPA patients had no perceptuomotor impediment to the performance of the task as shown by perfect copying scores. Incidental recall, indicative of the effortless (spontaneous) on-line encoding and subsequent reproduction of the items, was low for both types of items but more so for the words. This impairment was largely overcome during effortful on-line encoding as shown by the much higher scores at the end of the acquisition stage when compared to the incidental recall scores. Once the information entered on-line storage in a form that could sustain item reproduction, it was successfully transferred to off-line storage, as shown by the stability of performance when acquisition and recognition scores are compared. However, the PPA patients also showed a material-specific impairment in the retrieval of verbal items as shown by the declining performance when the recognition scores were compared to the delayed recall scores. In contrast, the DAT patients did not show consistent material-specific impairments and displayed abnormalities also at the stages of effortful on-line encoding (for non-verbal items) and retention (for verbal items).

Incidental recall, not commonly assessed in the clinical examination, tests the capacity for spontaneous online encoding and very short-term storage for purposes of immediate recall. The incidental recall condition used in this report can be considered to test a type of working memory but differs from standard working memory test methods in that the latter typically include the explicit instruction for recall. On this condition, patients with PPA could reproduce the shapes at a level close to controls and superior to the DAT patients. However, incidental word encoding was poorer than that of the controls and similar to that of DAT patients. This implies that verbal working memory, especially under conditions of automatic processing, is specifically impaired in PPA. In DAT, this type of short-term storage is impaired for both words and shapes.

This failure of verbal working memory was overcome by the Effortful Encoding trials where deliberate encoding resulted in successful on-line holding and immediate recall of both words and shapes. The DAT group required more trials than the PPA and control groups to reach criterion in the Effortful Encoding stage while the PPA group required more trials than the control group. All three groups were equally successful in the immediate reproduction of words and shapes through effortful encoding. However, the larger number of exposures required by the DAT patients to reach criterion and the lesser difference between scores of delayed recall and recognition in that group are consistent with a greater defect in the acquisition and retention of new information than in the PPA group.

A comparison of 3W3S performance between semantic and non semantic subtypes showed that the former group was more impaired for word recall than the latter but only in the delayed recall condition, perhaps consistent with the spread of atrophy to medial temporal areas in that group [17]. We conducted a previous study in which PPA patients viewed words and pictures in an incidental recognition memory task and gave abnormally high false positive response rates compared with cognitively normal individuals, primarily to semantically related words [18]. The number of target stimuli and distracters in that study was much greater than the 6 stimuli on the 3W3S task and this additional load may have contributed to the emergence of false-positive responses, a phenomenon that was not observed in the current study.

Although a number of studies have examined episodic memory in patients with various forms of progressive aphasia ([19–21]; also see [22] for a review and meta-analysis) none have made a direct comparison between the same number of verbal and non verbal stimuli within a single modality, namely, visual. Many studies have used standard tests that demand sophisticated language skills for comprehension of instructions. A number of case studies have documented the preservation of autobiographical memory but typically have required spoken responses. The Three Words Three Shapes Test therefore fills a gap for the need for a brief, convenient measure of memory that can be used even in moderate to severe stages of PPA.

One potential weakness of this study has to do with the nature of the stimuli. Created initially on the spot as a bedside test, the stimuli were not carefully controlled for imageability of words, verbalization of shapes, multiplicity of meanings for each word (e.g., "pride" and "station" have more than one meaning) and potential associations between the words and shapes. It was possible, then, that, for example, the pair including the word "Hunger" and a line drawing that looks like a mushroom could be recalled more easily than the others. We examined the data to determine if this had occurred and found only one example of incidental recall and one different example of delayed recall where the [Mushroom]-Hunger pair was the only one remembered. In all other instances where this pair was recalled, other pairs were also recalled. Thus, the word recall deficit in PPA overpowered any advantage offered by the implicit association between words and shapes.

There are a number of therapeutic implications from the results of the current study. First, on-line encoding, a necessary precursor for off-line storage and retention, was enhanced when the PPA patients were specifically asked to effortfully learn the information. This suggests that providing explicit encouragement to pay attention and multiple exposures (i.e., repetition of the information) for the purpose of remembering might enhance subsequent recall, even of verbal information. Another therapeutic strategy is to provide cues to aid retrieval. The PPA patients had no difficulties with recognition, suggesting that explicit cues can promote retrieval of information that might otherwise appear lost. For example, a "Communication Notebook", a collection of pictures, phrases, calendars, and other materials in a small format that a patient can use to activate information that is not quickly retrievable (http://www.brain.northwestern.edu/ppa/treatment.html) may provide benefical cues for retrieval.

The demonstration that a patient does not have a multimodal loss of new learning, and that the apparent memory loss is confined to an impairment of verbal retrieval, is critical for confirming the accuracy of the PPA diagnosis. The 3W3S test permits the objective demonstration of this pattern and contributes to our understanding of the intersection of memory and language.

## Acknowledgments

This study was supported by grants R01DC008552 from the National Institute on Deafness and other Communication Disorders, 5KL2RR025740 from the National Center for Research Resources, and by grant AG13854 (Alzheimer's Disease Core Center) from the National Institute on Aging. Disclosures of Funding from NIH and others.
